# Condiments

**Published:** 1873-08

**Authors:** 


### CONDIMENTS, such as pepper, mustard, and the like, are taken with the food, under the impression that they are "healthy," by promoting digestion, although it is not asserted that they have any nutritive value. It is claimed for them that they are beneficial, by promoting the flow of the saliva, gastric juice, and other fluids which are employed in digesting the food, and also that they promote that movement of the alimentary canal which is necessary to carry the refuse of what we eat downwards and outwards.

Taking it for granted that they do these things, it constitutes the strongest reason why a healthy person should not use them; because, if a man is well already, he cannot be better than well. In a state of health, nature throws out into the stomach all the gastric juice needed to digest that amount of food which is requisite for the needs of the system. Why compel her to throw out more?

If a man is in health the bowels act regularly, once in every twenty-four hours; oftener than that is disease. Why then use means to cause a greater than the natural motion?

If they promote the secretion of the natural juices of the stomach, if they do stimulate the intestines to more active movements, then they are good "medicines," but to be used only when there is a deficiency as to the two points named; and to that extent their tendency is to make a sick man well; on the other hand, they must as inevitably tend to make a well man sick—that is, to undermine his constitution, unless there are antagonistic influences in operation.

In proportion as they have the qualities claimed for them their use should be deferred, as something to fall back upon in case of sickness, such as indigestion, want of appetite and costiveness. All medicines lose their power by repetition or frequent use; and if their power for good is exhausted beforehand in health, there is nothing to fall back upon in disease. Exercise is healthful—over exercise is hurtful. This holds true as to every part of the human body—every muscle, every function, every gland. The eye requires a certain amount of watery fluid to enable the lids to work easily over the eye ball; but if it is stimulated to throw out more than is natural, that is inflammation, and inflammation is disease. So, if anything is taken into the stomach to excite an action greater than is natural, then there is excessive secretion, and that is disease. "Condiments," as they are called, stimulate the appetite; they tempt us to eat more than we otherwise would, and to that extent cause the stomach to be overtaxed and dyspepsia and kindred ailments follow. We all eat more of food that we consider "well seasoned" than if it were placed on the table in its simpler, natural state, cooked plain and well. All admit that "highly seasoned" foods are injurious—that means simply this, that a "little" seasoning, like liquor, at first satisfies us, but inevitably more and more is required, until at length nothing will answer. We cannot eat a meal unless we have the strongest mustard, the most fiery red pepper, and the sourest vinegar which can be obtained, and when that point is reached the man finds himself in a condition to be a regular liquor drinker, and falls an easy prey to bilious diseases, to apoplexy, or the intolerable gout.

Salt should not be considered a "condiment;" it is a nutrient, as it supplies to the system an element necessary to its healthful existence. Several of the "excretions" of the body have a saltish taste, as a tear drop, for example.

The blood has salt in it. Several of the domestic animals, the horse and the cow, take salt with avidity. If kept at a particular part of a field or pasture where cattle are kept, they will be noticed to quit feeding and go to the salt, and soon there will be a regular path marked out leading to the salt supply. And, as all nations and tribes of people use salt habitually, it may be well supposed that its use to man is natural and healthful.

